# The principles and promising future of sonogenetics for precision medicine

**DOI:** 10.7150/thno.98476

**Published:** 2024-08-12

**Authors:** Pengying Wu, Zhaoyou Liu, Wenxin Tao, Yubo Lai, Guodong Yang, Lijun Yuan

**Affiliations:** 1 Department of Ultrasound Medicine, Tangdu Hospital, Fourth Military Medical University, Shaanxi 710038, China.; 2 State Key Laboratory of Holistic Integrative Management of Gastrointestinal Cancers, Department of Biochemistry and Molecular Biology, Fourth Military Medical University, Xi'an, Shaanxi 710032, China.

**Keywords:** sonogenetics, ultrasound, biomolecular function, precision medicine, control, sonosensitive mediators.

## Abstract

Sonogenetics is an emerging medical technology that uses acoustic waves to control cells through sonosensitive mediators (SSMs) that are genetically encoded, thus remotely and non-invasively modulating specific molecular events and/or biomolecular functions. Sonogenetics has opened new opportunities for targeted spatiotemporal manipulation in the field of gene and cell-based therapies due to its inherent advantages, such as its noninvasive nature, high level of safety, and deep tissue penetration. Sonogenetics holds impressive potential in a wide range of applications, from tumor immunotherapy and mitigation of Parkinsonian symptoms to the modulation of neural reward pathway, and restoration of vision. This review provides a detailed overview of the mechanisms and classifications of established sonogenetics systems and summarizes their applications in disease treatment and management. The review concludes by highlighting the challenges that hinder the further progress of sonogenetics, paving the way for future advances.

## 1. Introduction

Recent advancements in control systems that enable direct manipulation of biomolecular functions through external stimuli show increasing promise for biological research and therapeutic applications 1-3. Various techniques, including chemical induction 4, optogenetics 5, radiofrequency radiation 6, and magnetogenetics 7, have been developed for an effective management of biomolecules. However, each method presents a unique set of challenges. Chemical inducers often lack precision in timing and location, leading to potential side effects 4. Optogenetics has limitations related to tissue penetration and typically requires the implantation of optical fibers for targeted applications 5. Radiofrequency radiation introduces ionizing effects, while magnetogenetics often relies on magnetic nanoparticles to transform magnetic fields into cellular signals, which may compromise specificity. Both radiofrequency and magnetic fields face general challenges related to specificity 6,7. Therefore, the development of a regulatory tool that is cell-type-selective, non-invasive, and capable of precise, tunable, and high-resolution control of biomolecular functions *in vivo* is of the utmost importance due to the above limitations.

Sonogenetics is a new ultrasound (US) approach that activates genetically encoded sonosensitive mediators (SSMs) in specific cell types, enabling the precise control of biomolecular functions with high spatiotemporal resolution 8. Thanks to the advantages of US, sonogenetics possesses a deeper penetration depth than visible light and a higher spatiotemporal precision than magnetic fields, thus overcoming the above limitations of optogenetics and magnetogenetics 9-11. Sonogenetics operates through two primary mechanisms: thermal 12 and mechanical 13. The mechanical aspect of sonogenetics exploits US-induced phenomena like streaming 14, cavitation 15, and acoustic radiation forces (ARF) 16 to activate SSMs, facilitating the remote manipulation of biomolecular functions. These mediators include mechanosensitive ion channels such as the two-pore potassium (K2P) family 17, the large conductance mechanosensitive (MscL) family 18,19, the Piezo family 20,21, transient receptor potential (TRP) channels 11, Prestin variant (N7T, N308S) 22, and gas vesicles (GVs) 23. The thermal effect of US, which induces hyperthermia typically ranging from 37 to 45 °C, modulates heat-sensitive transcription factors, genetic circuits, and thermosensitive transient receptor potential (TRP) cationic channels 12,24-27. Control is exerted through temperature-sensitive repressors (TSRs), such as TlpA 24 and TcI 25, or promoters, like heat shock promoters (HSPs) 26 and leftward and rightward promoters (PL-PR) 27. Sonogenetics is a promising approach for the precise regulation of biomolecular functions by integrating both thermal and mechanical gene control elements.

To date, sonogenetics has been successfully applied in various fields. This innovative technology provides a powerful, non-invasive means to manipulate both local and global neuronal functions, enabling the precise modulation of neurons on different brain regions with both high spatial and temporal resolution 28. Preclinical trials demonstrated the enhancement of specific behavioral outcomes like visual restoration 29, alleviation of Parkinsonian symptoms 30, and modulation of reward circuitry in the ventral tegmental area 31. Sonogenetics is used in tumor therapy to engineer thermal SSMs and US-controllable target cells for the targeted destruction of tumor cells through inducible therapeutic agent expression 32. Temperature-based transcription factors and genetic circuits are also engineered to convert the heat generated by US into genetic regulation, enabling the precise control over the expression of therapeutic agents 33. Furthermore, microbubbles (MBs) and GVs (unique air-filled protein nanostructures) can be used as sono-adjuvants to activate mammalian cells overexpressing SSMs through low-energy US 3. These systems sensitize the target cells and are widely used in several biomedical applications, enhancing the efficacy and scope of sonogenetics treatments.

**Figure 1:**
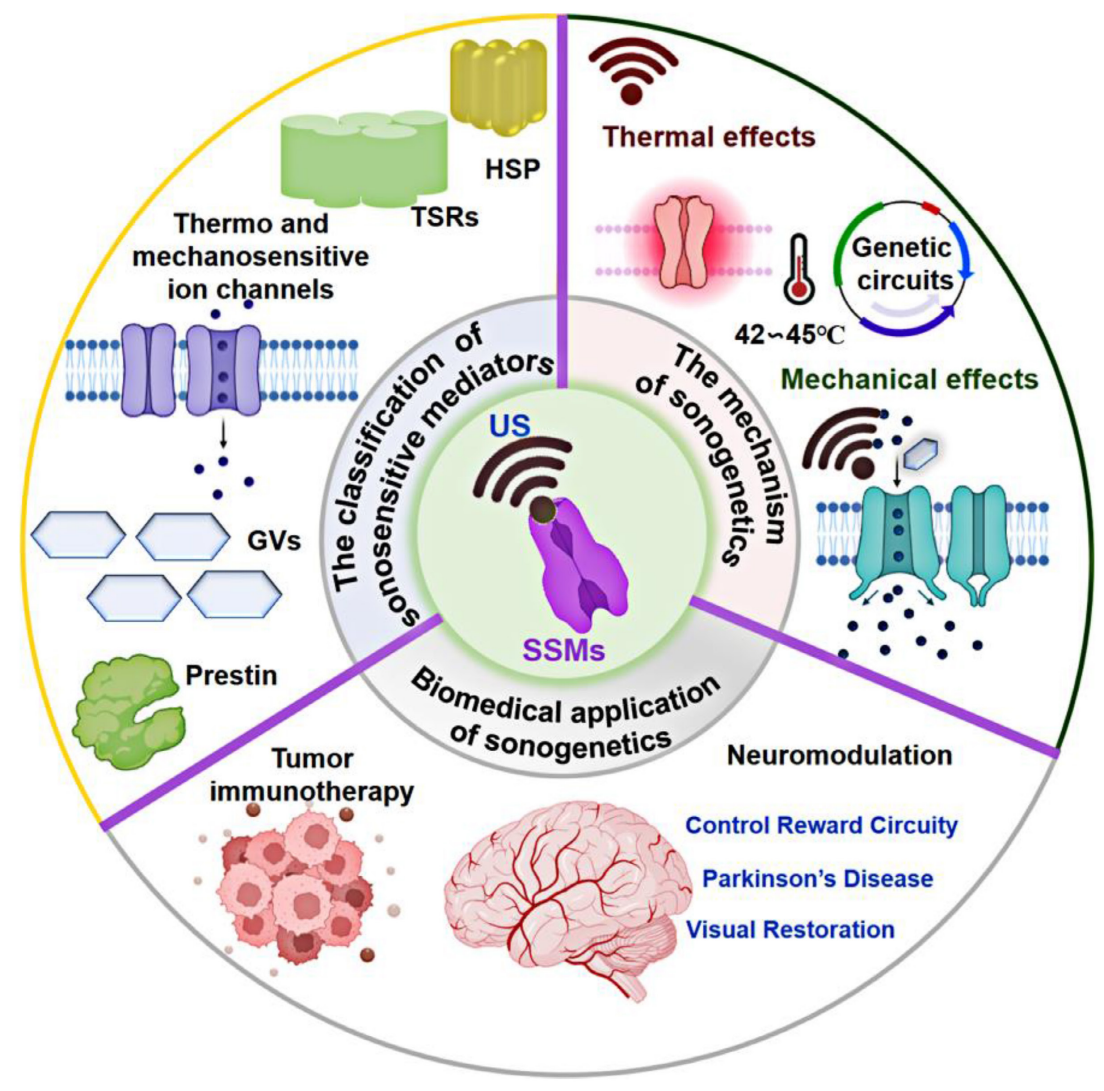
Schematics design of the classification, generation, mechanism, and biomedical application of sonogenetics. Created with BioRender.com.

This article offers a thorough and systematic review of sonogenetics covering its classification, generation, mechanisms, and ongoing challenges (as illustrated in **Figure 1**). First, it investigates the fundamental principles of US and explores their synergy in regulating cellular functions through sonogenetics. Next, it provides an overview of state-of-the-art biomedical applications of sonogenetics and discusses key considerations for a successful clinical application. By providing this background, this review aims to encourage innovative scientific strategies based on sonogenetics capable of addressing a broad spectrum of diseases.

## 2. Historical perspective and mechanistic basis of sonogenetics

### 2.1 History of sonogenetics

The initial exploration in the regulation of HSP70 through the thermal effects of US dates to 1998, with studies focusing on spatially controlling natural HSP70 expression *in vivo* 34. Based on this assumption, subsequent research demonstrated that coupling the HSP promoter with US induces the expression of luciferase genes, marking a significant advance. This significant development paved the way for using HSP promoters in conjunction with US to selectively express therapeutic genes in specific cell populations 35. In 2008, a separate research group reported that the mechanical effect of US enhances synaptic transmission by activating membrane channels 36.

**Figure 2:**
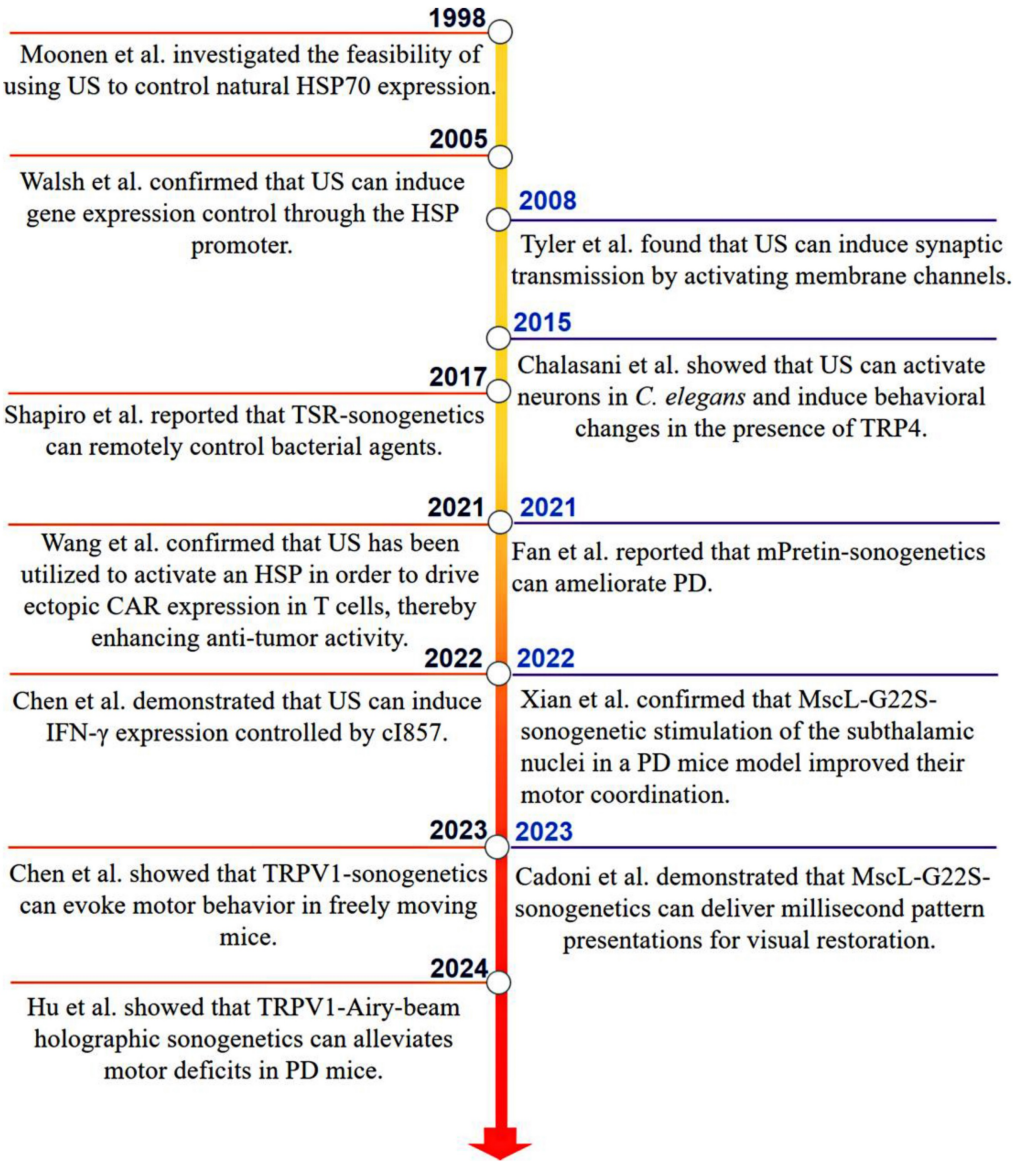
Key milestones in the development and application of sonogenetics have been achieved since the previous century, particularly in its biomedical applications. The term “sonogenetics” was introduced around 2015, involving the use of the mechanosensitive ion channel TRP-4 to alter the behavior of Caenorhabditis elegans worms in response to US 11.

Although these early studies suggested the potential of sonogenetics, a breakthrough occurred in 2015 when the Chalasani's team successfully activated neurons overexpressing the mechanosensitive ion channel TRP-4 using US. They demonstrated that US modifies the behavior of *Caenorhabditis elegans* worms in the presence of MBs, and coined the term “sonogenetics” to describe this innovative method 11. This pivotal study introduced sonogenetics to the wider scientific community, sparking a flurry of research activities. Subsequent proof-of-concept studies have explored a variety of SSMs for US activation, as shown in **Figure 2**.

### 2.2 Mechanisms of sonogenetics

**Figure 3:**
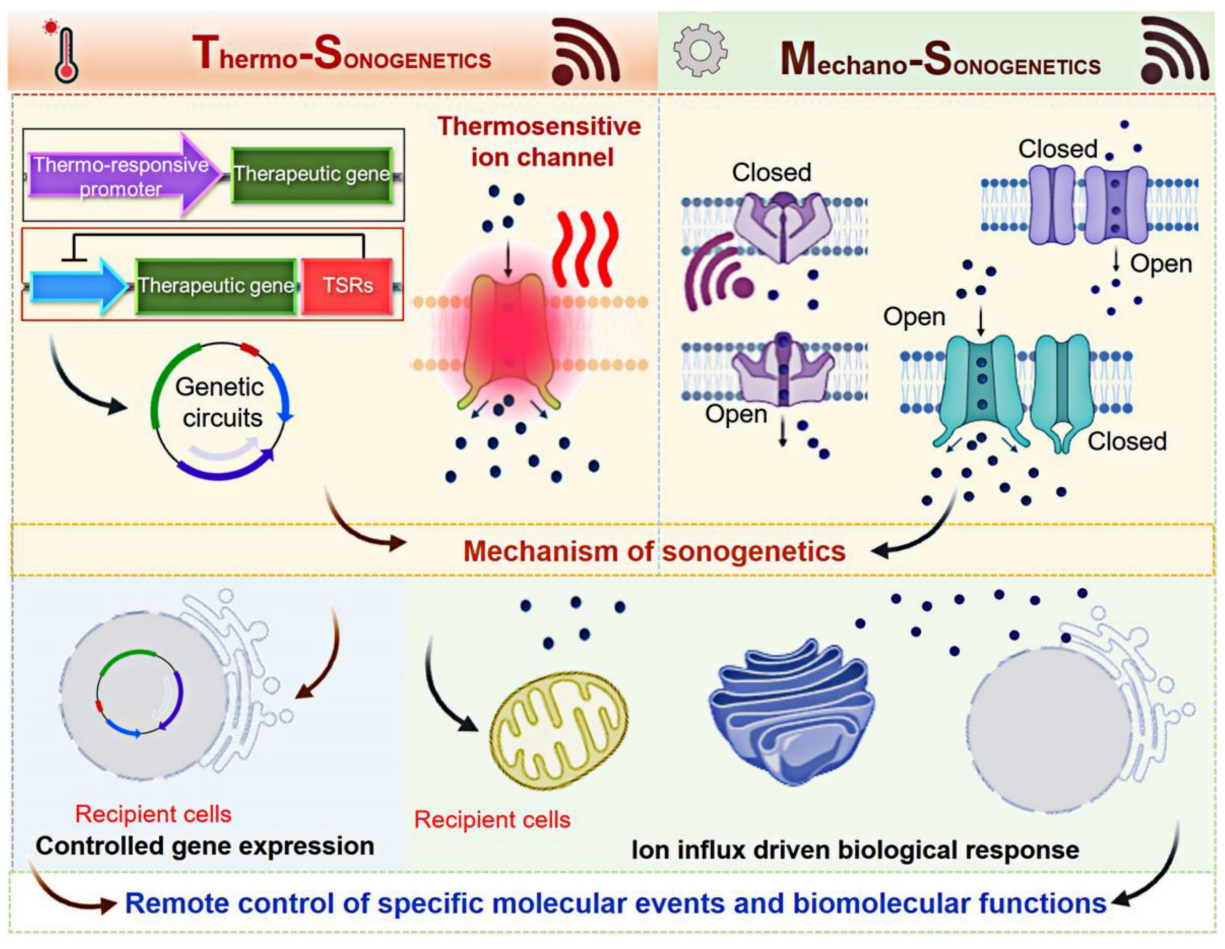
A scheme of mechanism of sonogenetics. Thermosensitive ion channels, temperature-sensitive repressors (TSRs), and heat shock proteins (HSPs) are utilized in thermo-sonogenetics, while mechanosensitive ion channels are employed in mechano-sonogenetics. These strategies ultimately enable the control of specific molecular events and biomolecular functions. Created with BioRender.com.

Sonogenetics uses both genetic engineering and US technology, representing a new avenue to control biomolecular function at the molecular level (**Figure 3**). The mechanism of sonogenetics involves two components: US waves and SSMs.

#### 2.2.1 Ultrasound waves

US is a type of mechanical wave capable of penetrating biological tissues across a range of frequencies, from sub-MHz to several MHz 37. Focused US (FUS) is a type of US that is spatially focused 38. Traditionally, US has been used for clinical imaging purposes due to its non-invasive nature and exceptional spatiotemporal resolution 39. Over the years, US has demonstrated a spectrum of biological effects influenced by parameters such as frequency, power, and duration that are typically classified as “thermal” or “mechanical” 40,41.

As regards thermal effect, US energy is dissipated to thermal energy as the acoustic waves propagate through the tissues and generate friction. FUS leads to localized tissue heating. At high intensities, continuous acoustic waves induce necrosis and thermal stress; in contrast, lower intensities may lead to sub-ablative hyperthermia, characterized by significant but non-lethal heat stress signals 42. US hyperthermia can be used to turn on thermal bio-switches. Specific examples of thermal bio-switches-mediated sonogenetics are described in this review.

On the one hand, US acoustic waves are propagated through an elastoviscous medium. This propagation actively or passively creates an acoustic pressure gradient based on standing waves, absorption, and tissue acoustic property gradients, and results in the generation of a non-zero net force on the medium, which is known as ARF 43. As a complement to the thermal effect, ARF applied to sonogenetics is used to induce mechanical signaling. This mechanical force combined with genetically encoded SSMs allows the control of cellular functions by sonogenetics 8. This review explores specific examples such as the modulation of the excitability of specific neurons and the induction of the release of therapeutic payloads from cells.

In some cases, US produces cavitation where MBs grow and collapse with compression and rarefaction. MBs undergo symmetrical linear oscillations at low acoustic pressures, while the oscillations become asymmetric under increasing acoustic pressures, promoting the expansion phase 44. The shear stress from oscillating MBs generates fluid micro-streaming-induced membrane tension variations that activate mechanosensitive ion channels 45. The MBs-US-induced changes in membrane permeability increase ion flow and lead to changes in membrane potential for control of cellular functions 46.

There are distinct advantages of using thermal and mechanical bio-effects to regulate cellular function, particularly at a level of safety in biological systems and precise spatiotemporal control.

#### 2.2.2 SSMs

Two categories of SSMs function as pivotal links between sonogenetics and cellular function regulation and have been identified across a diverse range of organisms from bacteria to mammals 47,48. Thermal SSMs are typically inactive under normal physiological conditions presenting intriguing possibilities. For example, the mammalian HSP70 is normally dormant but can be activated by mild hyperthermia (at 43 °C for a brief period) without causing significant tissue damage 49. Thus, the incorporation of HSP70 into gene circuits allows the precise control of the expression of therapeutic agents in mammalian hosts using sonogenetics. Another significant system involves TSRs, a group of thermal SSMs that regulate gene expression using the phage lambda promoters PL-PR. These systems inhibit gene expression under standard conditions and are only activated when the temperature exceeds 37 °C 50. The complex interplay of thermal-sensitive mediators in the framework of sonogenetics is further described in Section 3.

In contrast, mechanical SSMs are primarily represented by mechanosensitive ion channels. These channels have unique structures and mechanotransduction mechanisms that respond to US stimulation 51. It is widely recognized that US deforms cell membranes, indirectly causing conformational changes in channel structures or directly influencing channel components to the transition from a closed to an open state 52-54. Research has underscored the importance of GVs and the Prestin protein family in eliciting responses to sonogenetics, suggesting that these mediators enhance the activation of US-responsive mechanosensitive ion channels 55,56. The role of mechanical SSMs in the framework of sonogenetics is described in Section 4.

## 3. Thermo-sonogenetics

Rapid oscillations in pressure due to US waves, along with the associated cycles of mechanical loading and unloading, generate thermal effects in biological tissues. When applied at high intensity, US rapidly increases the temperature in a focal area from 55 to 80 °C 57,58. Low-intensity waves induce hyperthermia in targeted regions by carefully adjusting US parameters 59. Meticulous designs using these specific thermal advantages result in the creation of thermal-sensitive mediators and genetic circuits capable of effectively regulating biomolecular functions (**Table 1**).

### 3.1 HSP promoter-mediated sonogenetics

During febrile conditions, HSP promoters are widely used by the body to produce stabilizing proteins 64. HSP70 promoters are of particular value in gene therapy due to their robust ability to initiate and regulate the transcription of therapeutic genes. The inducible HSP70 promoter amplifies gene expression thousand-fold in response to hyperthermia 65. A study showed that the HSP70 promoter effectively controls transgene expression under localized hyperthermia conditions both *in vitro* and *in vivo* 66. Furthermore, magnetic resonance imaging (MRI) can be used to monitor US-induced hyperthermia to allow the precise modulation of therapeutic gene expression in a specific temperature range 67. However, transient US hyperthermia alone is insufficient for a sustained gene activation and cellular function. To overcome this limitation, the *Cre-lox* gene switch is incorporated into the HSP inducible system. The transient expression of Cre induced by US hyperthermia leads to the excision of the STOP cassette, enabling the switch from ZsGreen expression to the continuous production of the therapeutic agent 33. Traditional US hyperthermia inducible systems require the cloning of each therapeutic gene under the HSP promoter, which is a challenge in the control of the expression of multiple genes. To address this issue, a more flexible and efficient system has been developed to simultaneously modulate multiple therapeutic genes. This system uses US hyperthermia to control gene expression through an enhanced CRISPR-Cas system. Here, the Cas protein and its effector are regulated by a truncated HSP70 promoter that quickly responds to hyperthermia. A tailored guide RNA array is then used to specifically target one or more therapeutic genes, ultimately facilitating multiplexed activation 10. This breakthrough highlights the potential of sonogenetics as an innovative approach for enhancing disease treatment by precisely managing molecular events and cellular functions.

### 3.2 Sonogenetics mediated by TRP cationic channels

Thermosensitive ion channels provide a unique method for manipulating biomolecular functions using US hyperthermia by focusing specifically on the thermosensitive TRP family of channels 68. A study from the 1990s demonstrated that temperatures above 42 °C activate the TRP vanilloid 1 (TRPV1) ion channel, thereby influencing neuronal synaptic activity 69. Notably, this activation threshold is only slightly higher than the normal body temperature of most mammals. TRPV1 is thus characterized by inactivity at normal body temperature and activation at approximately 42 °C 70. In neurons that express TRPV1, the associated peak temperatures in the motor cortex of 38.5-39.7 °C also evoke activation 61. TRPV1 triggers a significant activation even at low expression, minimizing the risk of toxicity that comes with introducing foreign proteins 70. TRPV1 has been employed in genetics-based neuromodulation due to its unique properties. For example, TRPV1-based sonogenetics has been used to stimulate motor behavior by targeting the motor cortex in more superficial brain regions under US irradiation with appropriate parameters. Furthermore, this TRPV1 channel has also been used in recent research to control motor behavior in freely moving mice by targeting the striatum in deep brain areas 60,61.

### 3.3 TSR-mediated sonogenetics

At present, US-TSRs are primarily derived from transcriptional repressors found in bacteria and phages, particularly TlpA and TcI 24. TlpA is a self-regulator from the virulence plasmid of* Salmonella typhimurium* that features a C-terminal helix-helix domain that undergoes temperature-dependent unfolding between 37 °C and 45 °C. Its N-terminal DNA-binding domain remains in a low-temperature dimer state, inhibiting the transcription from 52-bp TlpA-operated promoters 71,72. Shapiro *et al.* reported the use of the engineered variant TlpA_36_ for the spatiotemporal control of microbial therapeutics through US. In this study, *Escherichia coli* expressing GFP under TlpA_36_ regulation was subcutaneously injected into the hindlimbs of nude mice, and MRI-guided US was applied at one site to maintain a local temperature of 41 °C for 45 min. This approach resulted in specific GFP expression at the targeted site *in vivo*, illustrating the potential of sonogenetics to manipulate cellular functions in biomedical applications 24.

Subsequently, the same research group reported another effective repressor capable of activation through sonogenetics. TcI is a temperature-sensitive mutant of the bacteriophage lambda protein “cI” that binds to its cognate operator sites in the tandem pR-pL promoter. Repression by TcI can be controlled by significant temperature shifts, with TcI and TcI42 exhibiting activation temperature thresholds of 38 °C and 42 °C, respectively 62. Additionally, the cI mutant cI857 can be used for temperature-sensitive transcription activation through sonogenetics. At lower temperatures, the cI857 repressor, which is expressed from the same vector, suppresses the transcription from the tandem pR-pL promoter. However, US-induced hyperthermia (45 °C) rapidly deactivates the cI857 repressor, enabling the transcription of therapeutic genes under the pR-pL promoter 63.

## 4. Mechano-sonogenetics

When US waves encounter reflectors or scatterers with different acoustic impedances, they create a physical momentum that converts sound waves into mechanical force impacting specific tissues 73. In the case of cavitation, the shear stress from MB oscillations generates fluid micro-streaming 45. These mechanical forces exerted by US alter the conformations of mechanosensitive proteins, in turn influencing cellular mechanics. This change triggers the activation of mechanosensitive proteins and induces subsequent signaling pathways 74. This process underpins the development of various mechanosensitive mediators designed to regulate biomolecular functions through sonogenetics (**Table 2**).

### 4.1 K2P ion channel-mediated sonogenetics

K2P channels are widely expressed in the human body and contribute to background potassium conductance in various cell types 79-81. Structured as dimers, each K2P channel consists of two subunits each containing four transmembrane helices (M1 to M4) and two pore-forming domains (P1 and P2) that interact to form a functional channel. The background conductance provided by K2P channels is essential for maintaining the negative membrane potential in cells, thereby reducing the excitability of excitable cells 82. K2P channels respond to various stimuli and molecules including internal and external pH, phospholipids, voltage, temperature, mechanical stretching of the membrane, interactions with the cytoskeleton, and intracellular signals. Notably, only the TRAAK and TREK channels in the K2P channel family are known as mechanically sensitive 83.

A study using the Xenopus oocyte system to express TREK-1, TREK-2, and TRAAK reported that the currents through these ion channels increased by an average of 23% following US stimulation. This enhancement suggests that sonogenetics modulates the activity of these ion channels, thereby influencing transmembrane currents 17. Further studies showed that US activates TRAAK ion channels expressed in the cortical neurons of mice, highlighting the potential of TRAAK channels for targeted neuromodulation in sonogenetics to specifically alter the activity of genetically defined cells 75.

### 4.2 TRP ion channel-mediated sonogenetics

TRP ion channel proteins, which are characterized by six transmembrane segments, are divided into six subfamilies (TRPC, TRPV, TRPM, TRPA, TRPP, and TRPML) based on amino acid sequence similarities 84. Known for their calcium permeability and diverse activation properties, these channels integrate various stimuli to modulate downstream cell signaling through ion influx and membrane depolarization. The TRP family features a broad array of gating mechanisms that include constitutively active channels as well as those gated by voltage, mechanical forces, temperature, and ligands 85.

Chalasani *et al.* reported that TRP-4 ion channels, which are crucial pore-forming subunits in mechanotransduction channels, are effectively activated through sonogenetics; however, this activation requires the presence of MBs, which limits its applicability in mammalian models 11. Subsequent studies have thus aimed to identify more responsive ion channels that do not depend on MBs. For example, low-intensity, low-frequency US stimulates endogenous TRPA1 ion channels *in vitro* 76. Furthermore, TRPC1, TRPP2, and TRPM4 in the motor cortex are activated through sonogenetics 13. Moreover, *in vivo* experiments showed that human TRPA1 expressed in the motor cortex is activated through sonogenetics 77.

TRPV1 is notable within the TRP family as an ion channel that is activated through US-induced hyperthermia, as discussed in Section 3.

### 4.3 MscL ion channel-mediated sonogenetics

MscL, which is found in *Escherichia coli*, is a pentameric channel composed of five subunits, each with two transmembrane helices (TM1 and TM2) and alkaline phosphatase fusion constructs. The TM1 helices from the five subunits cluster tightly to form the pore region, while the TM2 helices embedded in the lipid membrane sense mechanical forces and regulate MscL opening. The TM1 helix, which is highly conserved across MscL channels, is involved in channel gating. Mutations in this region significantly alter channel behavior, increase the mechanical sensitivity of MscL and enable the opening with a minimal membrane tension. Activated MscL channels demonstrate high electrical conductivity (3 nS), thereby facilitating the passage of ions, water, and small proteins 86,87.

Structural modifications to the TM1 and TM2 helices in MscL over time result in a variety of mutants with different levels of mechanical sensitivity 88. Unlike mechanosensitive ion channels in the K2P and TRP families, prolonged stimulation does not result in inactivation of MscL, making it suitable in the regulation of biomolecular functions through sonogenetics 83. Heterologous expression of MscL-G22S is mechanically sensitive and is activated by tethered MBs in retinal pigment epithelial (RPE) cells 89. Cells expressing MscL-G22S respond to sonogenetics stimulation both *in vitro* and *in vivo*, leading to Ca^2+^ influx and neuronal activation in areas expressing MscL 78. Similar to MscL-G22S, another mutant MscL-I92L also shows increased mechanosensitivity compared to the wild-type, making it an effective mediator of US-activated responses in primary cultured neurons 18.

Nevertheless, there are remaining issues on the large opening of MscL ion channels, potential interactions with other ion channels, and the tendency to aggregate into clusters 90, 91. Extensive opening allows the passage of ions as well as water and certain proteins, which reduces the mechanosensitivity of MscL when it forms clusters.

### 4.4 Piezo ion channel-mediated sonogenetics

Mechanosensitive Piezo ion channels are large transmembrane proteins including more than 2,500 amino acids characterized by a unique 38-transmembrane (TM) helix topology that forms a propeller-shaped trimeric structure. This structure features a central ion-conducting pore surrounded by three peripheral mechanosensing blades 92,93. The distinctively curved TM region of these blades creates a nano-bowl configuration that results in a substantial expansion of the in-plane membrane area, endowing the Piezo channels with exceptional mechanical sensitivity 94. A previous study showed that Piezo1 forms mechanically activated, non-selective cationic channels with a slight preference for Ca^2+^ over monovalent sodium ions (Na^+^) 92. Heterologous expression of Piezo1 ion channels is activated through sonogenetics in non-neuronal cells 14,95. For example, sonogenetics activates the heterologous expressed Piezo1 in T-cells, leading to calcium influx and subsequent expression of target genes 14. Furthermore, Shen *et al.* developed a Piezo1-targeted microbubble (PTMB) that binds to the extracellular domains of the Piezo1 channel in N2A cells and cultures of primary neurons. The use of these PTMBs facilitates the stimulation effect of US on neuromodulation 96. However, such activation typically requires high-frequency US or MBs.

### 4.5 Prestin family-mediated sonogenetics

It is well established that various species are able to detect US and echolocate within their environments. Prestin, an auditory-sensing protein found exclusively in outer cochlear hair cells, has been the subject of extensive *in vitro* research. Changes in transmembrane voltage induce conformational changes in Prestin, resulting in cellular contraction and elongation, while Prestin deficiency is associated with hearing loss in both humans and mice 97-99. Since Prestin is a vital component for high-frequency hearing in mammals, it may also be capable of sensing US-induced voltage fluctuations across membranes and converting them into mechanical actions through electrical mobility.

The Prestin mutants (N7T, N308S) demonstrate increased sensitivity to US *in vitro*, and subsequent studies confirmed their activation by US *in vivo* 30*.* Although a study supports the role of Prestin (SLC26a5) in electromotility and cochlear amplification when activated by US 100, the use of Prestin in sonogenetics requires careful consideration, especially regarding its role in mediating intracellular electromotility.

### 4.6 GVs as sono-adjuvants

GVs are naturally occurring, nano-sized, gas-filled protein structures in cyanobacteria and archaea that demonstrate a significant potential for US applications. Like MBs, GVs feature a gas core encased by a protein shell, enabling them to mechanically perturb the surrounding tissues through oscillations and/or ARF within the acoustic field 101,102. It has been confirmed that GVs effectively activate neurons when stimulated by US 56,103. A proposed mechanism for this effect is an amplification of US-activated mechanosensitive ion channels. The introduction of the mechanosensitive ion channel MscL-G22S into neurons enhances calcium influx and stimulates neuronal activity 103. Despite these promising findings, several challenges hinder the use of GVs for sonogenetics. Firstly, the effectiveness of GVs-based US activation depends on endogenous mechanosensitive ion channels whose functions are not yet fully understood. Secondly, like heat-responsive circuits, genetic expression of GVs in mammalian cells is complicated as multiple genes are required for their assembly 9.

## 5. Biomedical applications of sonogenetics

**Figure 4:**
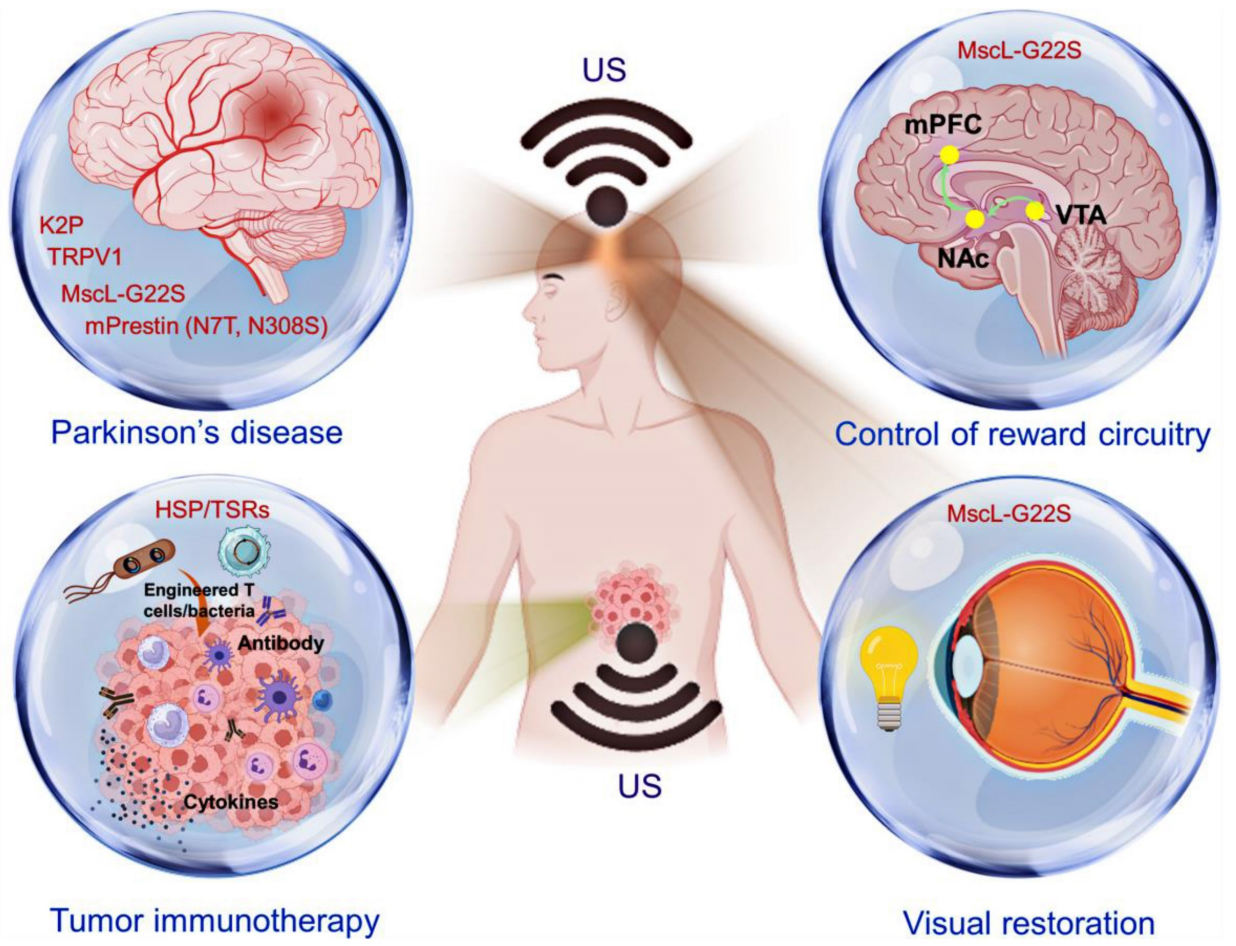
A schematic representation of the biomolecular functions regulated by sonogenetics in tumor modulation and neuromodulation. Introducing engineered T cells or bacteria has enabled several immune strategies to activate antitumor activities through sonogenetics 14,33,62,63. Conversely, by incorporating mechanosensitive ion channels, sonogenetics enhances specific behavioral outcomes, including visual restoration, mitigation of Parkinsonian symptoms, and modulation of reward circuitry 29,31,116,117. Created with BioRender.com.

Sonogenetics, which integrates SSMs and US, has emerged as a highly precise method for manipulating biomolecular functions in deep tissues. This section explores the biomedical applications of this innovative technique and highlights the latest advancements in the field of sonogenetics (**Figure 4**).

### 5.1 Tumor immunotherapy

Tumors exhibit a complex array of biological abilities, including sustained proliferative signaling, evasion of cell death, resistance to growth suppression, metabolic alterations, promotion of angiogenesis, and facilitation of invasion and metastasis. Tumors often recruit various seemingly normal cells to form the tumor microenvironment, which contributes to these hallmark abilities. As a result of these characteristics, traditional tumor treatments are frequently ineffective 104,105.

In recent years, cell therapies have emerged as promising and effective strategies for treating tumors. Within this context, sonogenetics offers a versatile approach to remotely and non-invasively modulate molecular functions, particularly in cell-based cancer immunotherapy. For example, a mechano-sonogenetics system has been used to remotely control cellular molecular functions 14. This innovative system involves coupling MBs to the surface of certain cells, such as Jurkat T-cell lines and primary T cells overexpressing the mechanically sensitive Piezo1 ion channel. The Piezo1 ion channels are activated upon US stimulation, leading to enhanced Ca^2+^ and subsequent activation of downstream pathways, including calcineurin activation, dephosphorylation of the nuclear factor of activated T-cells (NFAT) and its translocation into the nucleus. Subsequently, the translocated NFAT binds to the NFAT response element (NFAT RE 106) to initiate chimeric antigen receptor (CAR) expression. However, it is difficult to deliver MBs to the tumor site because of their limited half-life, resulting in restricted *in vivo* applications of MB-activated Piezo1 ion channels. Although calcineurin is calcium-sensitive, the downstream dephosphorylation substrate is not exclusive to NFAT, resulting in a challenge for accurately controlling NFAT dephosphorylation after US activation 107.

In a subsequent study, the same research group developed a thermo-sonogenetics system for controlling engineered T cell activation *in vivo* using clinically available MRI guidance. This system integrates the Cre-lox gene switch into an inducible sonogenetics system under the regulation of an HSP promoter. This system in Jurkat T-cell lines and primary T cells converts transient thermal stimulation into sustained CAR activation upon US stimulation 33. While the US stimulation and biocompatible HSP promoter induce a transient expression of various synthetic protein regulators, enhancing the safety of gene therapy and mitigating adverse host immune responses, there are two main challenges hindering the clinical application of this system. Firstly, engineered T cells face difficulties homing to tumor tissues following intravenous delivery. Secondly, the ability of T cells to penetrate and function in the immunosuppressive environment of solid tumors, particularly in hypoxic cores is limited 108-110. Thus, local injections were necessary to achieve effective therapeutic outcomes due to these challenges.

Immune activity in the tumor core sets the stage for a conducive microenvironment that attracts bacteria, facilitating the precise targeting of the tumor core post-systemic administration 111. Bacteria-mediated cell therapy has emerged as a promising approach to cancer treatment due to the intrinsic ability of bacteria to infiltrate tumors 112. The incorporation of sonogenetics promoter engineering and intelligent genetic circuits into bacteria offers a powerful means to finely regulate their function and behavior. For example, sonogenetics-activated engineered bacteria have been used to transiently induce US hyperthermia to trigger the sustained release of immune checkpoint inhibitors targeting CTLA-4 and PD-L1. By adapting TSRs TcI42 to the tumor-infiltrating probiotic species *E. coli* Nissle 1917 and designing PL-PR temperature-regulated gene circuits, it is possible to effectively control the expression of aCTLA-4 and aPD-L1. Fusion of CTLA-4 and PD-L1 antibodies with a PelB secretion tag has been used to enhance extracellular release of these agents upon US activation. In a study of experimental A20 tumor models, US-activated CTLA-4 and PD-L1 antibodies were successfully activated *in situ*, leading to significant suppression of tumor growth 62.

Further studies support the feasibility of spatiotemporal control over engineered bacterial function and activity through sonogenetics. Another research group 63 developed a system known as US-responsive engineered bacterium (URB) that is capable of regulating interferon-γ expression in response to US hyperthermia. By inserting the interferon-γ gene under the PL and PR promoters into *E. coli* MG1655, which specifically accumulates in hypoxic and necrotic tumor regions, the researchers precisely activate anti-tumor immune responses upon US irradiation. Sonogenetics enables the controlled production and secretion of IFN-γ in 4T1 tumor models by engineered *E. coli* MG1655, inducing apoptosis in 4T1 cancer cells, stimulating the polarization of M2-like macrophages towards an M1-like phenotype, and activating T lymphocytes (CD4^+^ and CD8^+^ T cells).

In conclusion, these findings highlight sonogenetics as a valuable tool for precise spatiotemporal control of biomolecular functions in tumor therapy.

### 5.2 Control of reward circuitry

Dopamine neurons in the ventral tegmental area (VTA) are crucial in the regulation of both adaptive and pathological brain functions associated with reward and motivation. As the principal source of dopamine for critical regions such as the medial prefrontal cortex (mPFC) and nucleus accumbens (NAc), these neurons significantly influence reward circuitry dynamics 113,114. Sonogenetics stimulation of MscL-G22S in the VTA effectively activates the mesolimbic pathway, leading to dopamine release in the NAc and influencing appetite regulation 31. Real-time place preference assay indicates that mice with MscL-G22S in their VTA dopamine neurons display increasingly aversive responses when FUS pressure is progressively increased from 0.04 MPa to 0.12 MPa. Fiber photometry has been used to pinpoint the reward circuits activated by the VTA. After stimulating the VTA with 0.3 MPa US, a rapid, synchronous increase in DA2m fluorescence was observed in the NAc of mice expressing MscL-G22S. These findings demonstrate that MscL-G22S-sonogenetics selectively induces dopamine secretion in neurons projecting to the NAc *in vivo*, thereby eliciting appetitive conditioning behaviors.

Such research sheds light on the complex mechanisms used by sonogenetics to modulate dopamine signaling in the mesolimbic pathway, providing new insights into the potential therapeutic applications of the modulation of reward-related behaviors and appetite regulation

### 5.3 Parkinson's disease

Parkinson's disease (PD) is the second most common neurodegenerative disorder in humans characterized by symptoms including tremors, bradykinesia, rigidity, and impaired balance 115. Various studies explored the potential of sonogenetics to alleviate Parkinsonian symptoms. One key *in vitro* study demonstrated the ability of sonogenetics to protect neuron cells from the toxic effects of 1-Methyl-4-phenylpyridinium (MPP^+^), a dopaminergic neuronal toxin. This protection was mediated by the activation of K2P ion channels, which trigger downstream pathways involving the phosphoinositide 3-kinase (PI3K)-Akt and ERK1/2 pathways. Although promising, such applications are yet to be applied to animal models of PD 116.

Another study showed that the overexpression of mPrestin (N7T, N308S) in dopaminergic neurons and subjecting them to focused US stimulation results in a significant increase in brain-derived neurotrophic factor (BDNF) and nerve growth factor (NGF) expression. These changes lead to a notable slowing of dopaminergic neuron degeneration and enhanced dopamine synthesis, resulting in a substantial recovery of motor function in PD model mice 30. Sonogenetics is also able to modulate neural activity in the subthalamic nucleus (STN) and enhance motor performance in PD mice 31. By injecting EYFP/MscL-G22S AAVs into the STN region of PD mice, researchers observed that the stimulation of MscL-expressing STN regions leads to an increased c-Fos expression in the STN. PD mice showed significant improvements in motor function following sonogenetics stimulation, as assessed by the rotarod and open-field tests, indicating that sonogenetics targeting MscL-G22S in the STN effectively alleviates motor symptoms.

Although sonogenetics holds promise in alleviating motor deficits in PD mice, existing technologies have limited its potential. Chen *et al.* recently introduced an Airy-beam holographic sonogenetics (AhSonogenetics) to alleviate motor deficits in mice with PD, addressing the challenge of simultaneously targeting dysfunction in multiple brain regions. By injecting TRPV1 AAVs into the dorsal striatum bilaterally in PD mice, they observed that the stimulation of TRPV1 through AhSonogenetics using the dual-focusing metasurface improved the immobility of PD mice 117.

Overall, these findings highlight the therapeutic potential of sonogenetics for improving motor function and the quality of life in individuals affected by PD.

### 5.4 Visual restoration

Retinitis pigmentosa is a major cause of blindness, leading to significant sensory impairment. Recent studies explored the potential to restore limited visual perception through retinal stimulation 118. A groundbreaking 2023 study demonstrated that the combination of MscL-G22S ion channels with unconventional high-frequency US stimulation rapidly activates retinal or cortical neurons with remarkable spatiotemporal precision and acoustic energy deposition conducive to vision restoration 29. Targeting MscL-G22S in rat retinal ganglion cells (RGCs) leads to a robust and sustained ON spiking responses to sonogenetics stimulation using a 15 MHz US frequency, with most RGCs exhibiting ultra-short latency responses. The activation of MscL-G22S in cortical neurons elicits responses with millisecond latencies and a spatial resolution of at least 400 µm in the x-y plane. Behavioral experiments showed that mice trained to associate visible-light stimulation of one eye with a water reward during water deprivation exhibit an anticipatory lick success rate surpassing that of non-transfected MscL-G22S mice following cortical sonogenetics stimulation, suggesting a level of light perception post-stimulation. Therefore, this new technology characterized by rapid response dynamics, high spatial resolution, and cell-type specificity represents a potential for the advancements in high-resolution visual restoration at the cortical level.

## 6. Summary and future directions

Sonogenetics is an emerging approach that integrates modular component SSMs with noninvasive US waves technology, providing a new strategy for deep tissue high-resolution and non-invasive therapy. Sonogenetics has promising applications not only in dissecting neural circuits but also in the noninvasive treatment of diseases, such as cancer and neuromodulation. Notably, other than cancer and central nervous system diseases, the range of diseases that might be treated with sonogenetics may be wide, including orthopedic disorders, anaphylaxis, metabolic disease, tissue engineering and regenerative medicine, and others 119-122, for example, this technology may be soon used to activate heart cells and insulin pumps: implanting SSMs in the human heart/pancreas and activating these cells with an external US device could revolutionize pacemakers and diabetes treatment. In the fields of tissue engineering and regenerative medicine, sonogenetics has the potential to modulate tissue maturation spatiotemporally, impact the microenvironment, and alter cellular programming, thereby enabling precise control over the fate of engineered cells and tissues 122. However, like all emerging technologies, there are ongoing challenges that need to be addressed to treat patients across a broader spectrum of diseases.

### 6.1 SSMs

SSMs are crucial for ensuring the precise selectivity of sonogenetics. Viral vectors are commonly used to genetically modify specific cells. However, this approach raises safety and cost concerns due to the risk of viral vector leakage into non-targeted cells, potentially causing unintended side effects. In neuromodulation, there have been cases of involuntary activation of the ascending auditory system by US in various species including cats, mice, guinea pigs, and humans 123-126. Such unintended activation leads to auditory confusion and off-target effects, particularly in neuromodulation applications. Moreover, the distribution of mediators in various brain regions through intracranial injections damages the healthy brain tissue and backflow of viral vectors along the inserted cannula can compromise gene expression.

Ideally, mediators should exhibit high sonosensitivity, enhance safety, and minimize tissue disruption during therapeutic procedures. Although some promising SSMs have been identified, it is crucial to determine which ones show both structural and functional compatibility with sonogenetics responsiveness. Refining the structure and function of these mediators and their mutants may enhance the potential for an efficient remote control of their activity *in vivo* through sonogenetics. For example, the incorporation of long and highly charged domains enables the efficient transfer of US mechanical force to the protein structure, and successfully realizes the use of different enzymes and switching “on” and “off” the protein activity 127. In addition, the use of mechanoluminescent nanotransducer to achieve sono-optical energy conversion also enhances sonosensitivity. For example, Wang *et al.* developed liposome nanotransducers to achieve efficient light emission upon US stimulation. These nanotransducers enable minimal invasive deep brain stimulation through a flexible, mechanoluminescent sono-optogenetic system 128. Furthermore, exploring non-invasive approaches for mediators, such as using US-induced cavitation effects for blood-brain barrier disruption in specific brain regions and FUS-mediated MB destruction in targeted areas, is also of great promise 129-131. Future research should combine new minimally invasive drug delivery techniques with SSMs-mediated sonogenetics in small and large animal models to achieve a precise, spatially targeting, and cell type-specific regulation of function and activity.

### 6.2 US waves

The use of US waves in sonogenetics presents unique challenges, as this requires high spatial resolution and precise control of thermal and mechanical effects. Although high spatial resolution is achieved through high-frequency US (at least 400 µm in the x-y plane at 15 MHz 29), this comes at the expense of a reduced tissue penetration. For example, high-frequency US (> 10 MHz) is tightly focused to a small volume, but may not effectively penetrate deeper brain structures 77. Selecting the optimal frequency is thus crucial. Although current approaches are suitable for rodent models, designing a transducer array for nonhuman primates is essential for enhancing both the spatial resolution of acoustic stimuli and the penetration of US waves.

To achieve desired sonogenetics effects while minimizing unintended tissue damage or adverse effects, precise adjustment of US parameters (e.g., frequency, intensity, duration, and waveform) is essential. To date, most studies focusing on the relationship between US parameters and induced biological effects focused their attention on a single type of tissue or cell. Notably, tissues or cells with different acoustic properties may respond differently to sonogenetics stimulation. Therefore, the comprehensive exploration of the key factors influencing the biomedical effects of sonogenetics is crucial to optimize the potency of sonogenetics and guide the development of disease therapy.

The development of user-friendly interfaces for sonogenetics instruments tailored for clinical use is of utmost importance. An ideal sonogenetics device should enable easy adjustment of US parameters, precise acoustic positioning, real-time temperature monitoring, mediator delivery, and seamless integration of clinical diagnostics. Emphasis should be placed on precise parameter control to ensure safety, accounting for factors such as tissue characteristics, cavitation, and acoustic streaming. Thus, maintaining the controlled thermal and mechanical effects of sonogenetics on various tissues and cells is essential to accelerate putative clinical conversion.

## Figures and Tables

**Table 1:** Summary of thermal effect-based sonogenetics and their recent applications.

Mediators	US equipment	Operational methods	US parameters	Application	Tempera-ture	Ref.
TRPV1	The US equipment was customized by Xinxiang Chiyu Ultrasonic Equipment Co. LTD and consists of a signal generator and a power amplifier.	US-stimulated M1 region of the mouse brain under anesthesia using a collimator.	1.8 MHz, Stimulation 2 s and stop for 2 s, Duration: 400 s, Power: 6W.	Induction of calcium influx, Neuromodulation in the M1 region of mouse brain.	42 °C	60
	A specialized wearable FUS device was designed, featuring an FUS transducer and a base plate	Attachment of FUS transducer base plate to the mouse skull.	1.5 MHz, 40% DC, PRF 10Hz, Duration: 15 s 0.7 and 1.1 MPa.	Neuromodulation of motor cortex in freely moving mice.	0.7 MPa: 38.57 ± 0.31 °C 1.1 MPa: 39.69 ± 0.59 °C	61
HSP	The US field is generated using an unfocused air-backed transrectal transducer, with power delivery managed by a multichannel RF driving system.	A transrectal US transducer surrounded by a water-filled latex membrane is placed in the animal rectum.	1.5 MHz, Power levels reaching as high as 70 W.	Control of exogenous luciferase expression in canine prostate.	42 °C	35
	The FUS system consists of a 1.5 MHz 8-element annular array transducer, a 16-channel broadband radiofrequency generator, an X-Y positioning stage, and a degassing and water circulation system.	The FUS transducer is placed directly above the targeted region on the mouse's hindlimb. Thin layers of SCAN ultrasound gel are applied at both the skin-transducer and skin-bed interfaces.	Three pulses of 5 min heating by MRI-guided FUS.	Regulation of CAR-T cell activity in tumors using a *Cre-lox* gene switch.	43 °C	33
	The therapeutic array, operating at 1.5 MHz and comprising 128 elements, was sourced from Imasonic. An imaging array is employed for image guidance and treatment planning.	The needle thermocouple is inserted subcutaneously between the tumor and the body wall. After placement, the animal is positioned laterally with the tumor oriented towards the therapeutic array.	1.5 MHz 128-element, 0-80% DC, Duration: 15 min, Maximum intensity Ispta of 20 W/cm^2^.	Multiplexed genome regulation in Jurkat T cells and primary T cells.	43 °C	10
TSR-TlpA_36_	The FUS procedure utilizes a 16-channel ultrasound generator, a motorized MRI-compatible transducer positioning system, and an annular array transducer operating at 1.5 MHz.	FUS is used to activate subcutaneously injected bacterial agents at a specific anatomical site.	1.5 MHz, 41 °C for 45 min to 1 h	Activated engineered *E. coli* expressing GFP at a specific anatomical site.	41 °C	24
TSR-TcI_42_	A closed-loop thermal control system was developed to maintain temperature by adjusting the intensity of the FUS. A Velmex BiSlide motorized positioning system is utilized to submerge and position the 0.67 MHz FUS transducer. A signal generator produces the thermal ultrasound signal, which is subsequently amplified and transmitted to the ultrasound transducer.	Mice were anesthetized and secured in a vertical nose-up position to an acrylic arm connected to a manual 3D positioning system, facilitating three-dimensional translation of the mouse within the water bath. The target flank was then activated using the FUS system.	0.67 MHz, 50% DC, 0.6 MPa, A total of 1 h at 43 °C with 5 min pulse duration.	Activated engineered *E. coli* Nissle 1917 for the sustained release of aPD-L1 and aATLA-4.	42 °C	62
TSR-cI857	No specific description.	The ON/OFF US irradiation procedure was adjusted to keep the temperature constant, and the mice were irradiated with the ultrasound for 30 min.	0.96 MHz, 150 ms pulse period, 100 ms pulse width, 3s ON and 5s OFF, 3.52 MPa.	Activated engineered *E. coli* MG1655 expressing IFN-γ for tumor immunotherapy.	45 °C	63

**Table 2:** Summary of mechanical effect-based sonogenetics and their applications.

Family	Mediators	US equipment	Operational methods	US parameters	Application	Ref.
K2P	TRAAK TREK-1 TREK-2	A tone burs US wave is generated using an immersion focus ultrasonic transducer, and this transducer is driven using a signal generator connected to a broadband amplifier.	Cells are positioned either on a plastic plate or within an aperture between two thin sheets of borosilicate glass at the focal point of the US focus.	10 MHz, 0.3-4.9 W/cm^2^.	TRAAK and TREK-1/2 sonogenetics facilitate the modulation of transmembrane currents.	17
	TRAAK	A US wave is generated using a focused-immersion ultrasonic transducer. A function generator is used to trigger the transducer's US pulses.	Inside-out patches excised from either oocytes or proteoliposomes are quickly transferred to the US chamber.	5 MHz, 0.2-3.6 W/cm^2^, Duration: 10 ms.	US activates TRAAK ion channels expressed in the cortical neurons of mice.	75
TRP	TRP4	An arbitrary waveform generator triggers a submersible transducer with a 300-W amplifier.	Anesthetized animals are surrounded by a solution of MBs and stimulated using US peak negative pressures.	0.69-3 MHz, Duration: 10 ms, 0-0.9MPa.	TRP-4-sonogenetics stimulation in PVD sensory/AIY neurons reverses the behavior of MBs-bound *C. elegans*.	11
	Mouse TRPA1	US is generated by two function generators connected in series, with the first function generator triggering the operation of the second. The bursts of pulsed sine waves produced by the function generators are amplified by a linear power amplifier and subsequently delivered to a single-element focused US transducer.	The US transducer is affixed above the mouse head, and the focus of the transducer is positioned to target the area of the motor cortex.	0.35-0.5 MHz, 50%DC, Duration: 100 ms, PRF 1.16, 1.5 and 2 kHz, >0.01MPa.	TRPA1-sonogenetics stimulation in astrocytes induces glutamate release for tail movement.	76
	*hs* TRPA1	US waves generated from custom-made, single-crystalline 127.68 Y-rotated X-propagating lithium niobate transducers are regulated by a waveform generator, with pressure control achieved through a 300-W amplifier.	*hs*TRPA1 is expressed in the left motor cortex of Npr3-Cre transgenic mice and controlled by US stimuli.	7 MHz, Duration: 100 ms, 0.15-2.5 MPa.	*hs*TRPA1 enables sonogenetic activation of mouse layer V motor cortex neurons *in vivo.*	77
	TRPC1, TRPP2, and TRPM4	The transducer is immersed in degassed water and oriented at a 20° angle relative to normal incidence on the Mylar film using a specialized holder. An Axon Digidata 1550 acquisition system is employed to program and produce a specified number of trigger pulses, which are subsequently transmitted to an arbitrary waveform generator. The output from the waveform generator is then amplified by a linear amplifier and utilized to drive the transducer.	Cultured primary murine cortical neurons are placed on an acoustically transparent mylar film at the top of a water tank, with a FUS transducer submerged in degassed water beneath them. The transducer uniformly delivers ultrasound to the neurons.	0.3 and 0.67 MHz, PRF 1 and 1.5 kHz, Duration: 500 ms, 0-15W/cm^2^.	Stimulation of TRPC1, TRPP2, and TRPM4 with US in primary cultured cortical neurons induces calcium influx and lowers the threshold for US activation.	13
MscL	Mscl-G22S	US equipment consists of a commercial transducer, two function generators, and a power amplifier to produce burst pulses.	Mice are anesthetized, US gel is applied to the shaved head, and the transducer is placed in contact with the gel.	0.5 MHz, 40%DC, PRF 1kHz, 0.05-0.5 MPa.	Induction of Ca^2+^ influx. Activation of neurons *in vitro*. Evoking electromyogram responses *in vivo*.	78
		US transducer is driven by a set of function generators and power amplifier generating pulsed US.	A wearable US transducer is mounted on the adaptor attached to the skull, coupled with US gel.	0.5 MHz, PRF:1KHz, Interval: 3s, 0.04-0.35 MPa.	Control of reward circuitry and improved motor coordination in PD mice.	31
		A TiePie Handyscope is used to produce the stimulus waveform, which is then passed through an 80 dB RF power amplifier connected to the transducer.	*In vitro*: US transducers are coupled with a custom-made coupling cone filled with degassed water and mounted on a motorized stage placed orthogonally above the retina. In vivo: US transducers are coupled to the brain with a custom-made coupling cone filled with degassed water and US gel on a motorized stage.	50% DC, PRF 1kHz, 0.5 MHz: 0.11 - 0.88 MPa, 2.25 MHz: 0.3 - 1.6 MPa, 15 MHz: 0.2-1.27 MPa.	Stimulation of retinal or cortical neurons with MscL-G22S sonogenetics occurs in milliseconds, with acoustic energy deposition compatible with vision restoration.	29
	MscL-I92L	Surface-acoustic-wave-based chips are used to generate US, and the transducers are driven using an arbitrary waveform generator connected to a broadband amplifier.	Cells are transferred to the chamber in the US device.	29.92 MHz, Duration: 50-400 ms, 0.12-0.45 MPa.	MscL-I92L- sonogenetics stimulation of rat primary hippocampal neurons evokes the spikes.	18
Piezo	Piezo1	The ultrasonic transducer is affixed to a three-dimensional mechanical stage to enable precise control over its positioning. A pulser/receiver and an oscilloscope are employed to align the natural focal point of the ultrasonic transducer with the target cell.	A glass tube is used to contain cells, and the ultrasonic transducer is positioned at a 45° angle for the glass surface for ultrasound stimulation.	2 MHz, 1%DC, PRF 100Hz, Duration: 10 min, Vpp: 22.12-31.6 V.	Piezo1-sonogenetics stimulation of Jurkat T cells and primary T cells induces anti-CD19 CAR.	14
Prestin	mPrestin (N7T, N308S)	The US transducer is driven by a function generator and a radio frequency power amplifier to transmit the US pulses.	The mice are immobilized using a stereotactic frame and anesthetized throughout the experiment. US is guided by 25-MHz US imaging and delivered transcranially to the brain of the mice.	0.5MHz, 10%DC, PRF 10Hz, Duration: 3s, 0.5 MPa	mPrestin-sonogenetics- based neuromodulation can alleviate Parkinson's disease symptoms	30
